# Induction of the Abscopal Effect with Immunotherapy and Palliative Radiation in Metastatic Head and Neck Squamous Cell Carcinoma: A Case Report and Review of the Literature

**DOI:** 10.7759/cureus.4201

**Published:** 2019-03-07

**Authors:** Ashwin Shinde, Jennifer Novak, Morganna L Freeman, Scott Glaser, Arya Amini

**Affiliations:** 1 Radiation Oncology, City of Hope National Medical Center, Duarte, USA; 2 Medical Oncology, City of Hope National Medical Center, Duarte, USA

**Keywords:** head and neck cancer, palliative, abscopal effect, quad shot, immunotherapy, ctla-4, pd-1 inhibitor, radiation

## Abstract

The induction of the abscopal effect using immunotherapy and radiation is under investigation through case reports and institutional studies. We describe a case of the abscopal effect with a combination of ipilimumab, nivolumab, and palliative radiation, in a patient with metastatic head and neck squamous cell carcinoma (mHNSCC).

## Introduction

Metastatic head and neck squamous cell carcinoma (mHNSCC) is a difficult disease to treat, with poor prognosis and median survival of between 6 and 12 months [1]. Standard therapy over the past decade has been a combination of cetuximab, cisplatin, and 5-flurouracil, albeit with significant toxicity and response rates of approximately 33% [2]. More recently, there has been significant interest in the use of immunotherapy in the management of mHNSCC [3]. Treatment with immunotherapy introduces discussion about the potential for the abscopal effect when combined with radiation. The abscopal effect was first described in the 1950s, and is seen with radiation, where a reduction in disease occurs outside the radiated site, through primarily immune mediation [4]. The most well-known case report of the abscopal effect was in a melanoma patient receiving ipilimumab who stopped responding after years of stable disease and developed progressive disease. The patient received palliative radiation, and subsequently showed partial responses in both the irradiated and unirradiated lesions [5]. Since then, there has been significant interest in combining immunotherapy with radiation to induce the abscopal effect, initially in mice, then across multiple disease sites [6-7]. We present a case of the abscopal effect observed in a patient with mHNSCC.

## Case presentation

**Figure 1:**
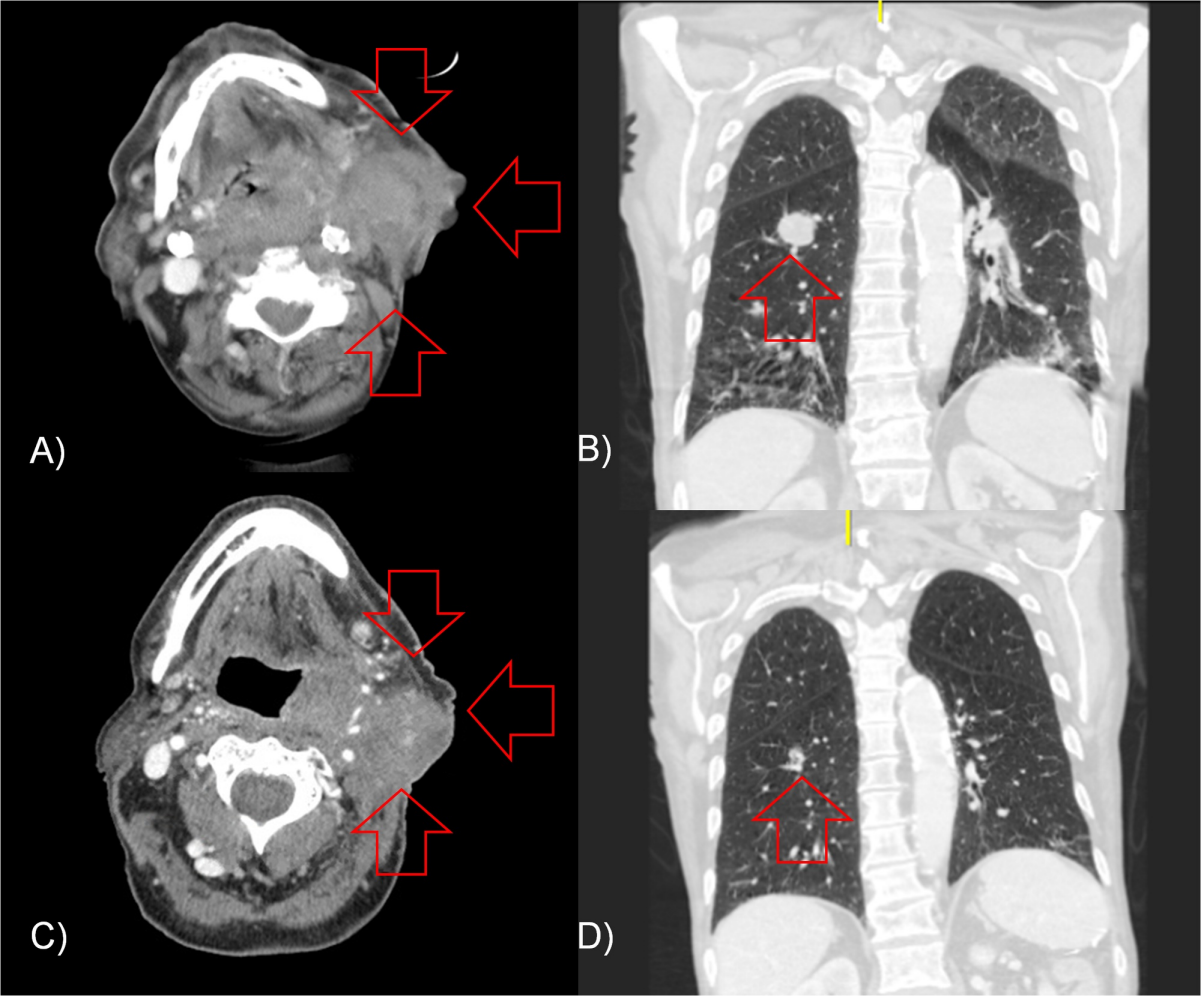
Neck and chest imaging before and after QUAD-SHOT with immunotherapy. CT imaging of the patient demonstrating (red arrows) (A) axial images of his primary disease and left neck adenopathy, and (B) coronal images of his largest pulmonary nodule, along with treatment response two weeks after completing QUAD-SHOT radiation treatment in the (C) axial and (D) coronal planes.

A 75-year-old male former smoker with 45-pack year history of tobacco abuse initially presented with swelling of the left neck, followed by progressive hoarseness and dysphagia over a course of one to two months. A biopsy of the left neck node demonstrated squamous cell carcinoma (SCC), p16 positive. The patient also had a CT scan and follow-up positron emission tomography-CT (PET-CT) showing a 4.5 cm x 7 cm mass involving the left hypopharynx and oropharynx, crossing midline and causing narrowing of the hypopharyngeal and supraglottic airway. There was also bulky left level II neck lymphadenopathy measuring up to 7 cm in diameter. The patient was also found to have three fluorodeoxyglucose (FDG) avid lung nodules as well, one of which was subsequently biopsied, confirming metastatic p16 positive SCC. The patient required tracheostomy and gastrostomy placement for threatened airway obstruction and dysphagia, respectively. He was initially enrolled on a clinical trial comparing first-line EXTREME chemotherapy versus ipilimumab and nivolumab, and was randomized to combination ipilimumab and nivolumab delivered concurrently every three weeks for four cycles. Follow-up imaging at eight weeks showed progression of disease, with growth of the primary and neck masses ulcerating through his skin (Figure 1A), as well as disease progression in the chest, with growth of previous nodules and development of additional pulmonary nodules, the largest measuring 2.4 cm (previously 1.6 cm, Figure 1B).

The patient had worsening symptoms associated with mass effect in the neck and was re-evaluated by Radiation Oncology at the request of the treating oncologist. He was treated with ‘QUAD SHOT’ for palliation [8], delivering 3.7 Gy BID x 2 days (total dose 14.8 Gy) to gross disease, and 3.3 Gy BID x 2 days (13.2 Gy) to microscopic areas at high risk for disease, including one nodal echelon beyond gross disease on the left (levels IB-V), and contralateral levels II-IV. No radiation was delivered to his lung disease. The patient continued through the third and fourth cycles of nivolumab and ipilimumab without breaks or delays. Radiation was delivered during the three-week time frame between immunotherapy cycles, and he tolerated treatment well with minimal pain and noticeable improvement in the left neck mass.

On follow-up CT scan at two weeks after completion of radiation, the primary and bulky left neck adenopathy had decreased in size by approximately 25% (Figure 1C). The metastatic pulmonary nodules had also all decreased in size with the largest nodule decreasing from 2.4 to 1.3 cm, approximately 50% (Figure 1D). His QUAD-SHOT regimen was repeated one month after initial treatment, as described above. Repeat imaging showed continued decrease in size of all neck and most lung disease, with one lung nodule minimally increasing in size. The patient received a third round of QUAD-SHOT as described above, with near complete resolution of his left neck mass. At 16 weeks from first round of radiation treatment, the patient had a continued response in his pulmonary metastases, followed by disease stability. At 20 weeks from start of radiation, one of his lung lesions had increased in size while the remaining lesions were either stable or decreased in size. At the time of this writing (10 months from initial diagnosis), he has completed a total of 14 cycles of ipilimumab and nivolumab on trial and is currently living with stable, asymptomatic disease.

## Discussion

This case represents, to our knowledge, the first reported case of the abscopal effect in mHNSCC. Our patient did not have an initial response to combination ipilimumab and nivolumab immunotherapy after four cycles of therapy. He received palliative treatment using QUAD-SHOT, a high dose per fraction treatment that provides short treatment times, acceptable toxicity, and ability to repeat treatment, dependent on patient’s prognosis and level of symptomatic relief [8]. After this course of palliative radiation, the patient showed partial response in both the irradiated neck and the unirradiated chest.

 No previous cases of the abscopal effect in mHNSCC have been reported in the literature. Objective response rates (ORRs) with immunotherapy alone in mHNSCC are low. When used as second-line therapy with progression on platinum-based chemotherapy, single agent nivolumab has an ORR of 13.3% [9], single agent pembrolizumab has an ORR of 18% [10], and single agent durvalumab has an ORR of 6.5%-16.2% [11-12]. Combination therapies evaluated include durvalumab and tremelimumab (ORR 7.8%) [13]. While the results of Checkmate-141 [9] showed improved survival compared to second-line single-agent chemotherapy in a phase III setting, additional studies are required to determine whether immunotherapy can lead to better survival than combination cisplatin, cetuximab, and 5-flurouracil as first line therapy for mHNSCC.

Results from currently ongoing trials may assist in future management decisions. KEYNOTE-040 will evaluate pembrolizumab in a phase III fashion against second-line single agent chemotherapy (Clinicaltrials.gov #NCT02252042). Another trial is evaluating second-line cyclophosphamide, avelumab, and single fraction radiation of 8 Gy in mHNSCC [14]. A French group is evaluating combining durvalumab and tremelimumab with stereotactic body radiation (SBRT) in mHNSCC patients [15]. Additional studies evaluating the role of immunotherapy in mHNSCC include combining SBRT with nivolumab (Clinicaltrials.gov #NCT03539198), comGiven poor ORRs of systemic immunotherapy alone in mHNSCC, lack of other acceptable treatment options, and poor prognosis of this patient population, we present this case report primarily for hypothesis generation; clinicians may consider combining immunotherapy with radiation in mHNSCC in an attempt to improve ORRs and potentially improve oncologic outcomes.bining nivolumab with experimental medication BMS-986205 and comparing it to cisplatin, cetuximab, and 5-fluorouracil (Clinicaltrials.gov #NCT03386838), combining pembrolizumab with clopidogrel (Clinicaltrials.gov #NCT03245489), and combining pembrolizumab with afatinib (Clinicaltrials.gov #NCT03695510).

Given poor ORRs of systemic immunotherapy alone in mHNSCC, lack of other acceptable treatment options, and poor prognosis of this patient population, we present this case report primarily for hypothesis generation; clinicians may consider combining immunotherapy with radiation in mHNSCC in an attempt to improve ORRs and potentially improve oncologic outcomes.

## Conclusions

Our patient exhibited evidence of the abscopal effect when doublet immunotherapy did not induce a response, palliative radiation was added, and the combination led to partial response in both irradiated and unirradiated sites of disease. We feel that this case report is hypothesis generating for possibly combining immunotherapy and radiation to improve objective response rates and other oncologic outcomes in patient’s mHNSCC.
